# Soil quality index: Exploring options for a comprehensive assessment of land use impacts in LCA

**DOI:** 10.1016/j.jclepro.2018.12.238

**Published:** 2019-04-01

**Authors:** Valeria De Laurentiis, Michela Secchi, Ulrike Bos, Rafael Horn, Alexis Laurent, Serenella Sala

**Affiliations:** a European Commission, Joint Research Centre, Via Enrico Fermi 2749, I-21027, Ispra VA, Italy; b University of Stuttgart, Insitute for Acoustics and Building Physics, Department Life Cycle Engineering, Wankelstrasse 5, D-70563, Stuttgart, Germany; c Technical University of Denmark (DTU), Department of Management Engineering, Division for Quantitative Sustainability Assessment (QSA), Lyngby, Denmark

**Keywords:** Land occupation, Land transformation, Life cycle impact assessment (LCIA), Soil quality index, Normalisation, Regionalisation

## Abstract

Impacts associated with land use are increasingly recognized as important aspects to consider when conducting Life Cycle Assessment (LCA). Across the existing models accounting for land use activities in life cycle impact assessment, a balance is yet to be found between complexity and comprehensiveness on one hand, and applicability on the other hand. This work builds on the LANd use indicator value CAlculation (LANCA^®^) model, assessing the impacts of land use activities on five soil properties, and aims at developing an aggregated index to improve its applicability. First a statistical analysis is conducted, leading to the shortlisting of the four most significant soil quality indicators. Then two options for aggregating the selected indicators are presented: the soil quality index (SQI), based on linear aggregation, and the normalisation–based soil quality index (NSQI), where the aggregation process involves normalisation integrated into the characterisation step. Country-specific and global average characterisation factors (CFs) are calculated for 57 land use types considering both land occupation and land transformation interventions with the two suggested approaches. The two indices present similar ranking of land use types but the relative contribution of the separate indicators to the aggregated index varies according to the approach adopted. The differences between the aggregation approaches suggested are discussed, together with the limitations related to both the LANCA^®^ model and the aggregation approaches. This work represents a first step towards the widespread application of a comprehensive and robust land use model at midpoint level in LCA. Finally, a number of recommendations for the future development of the LANCA^®^ model and of the related soil quality models are provided.

## Highlights

• Soil quality needs to be evaluated considering different drivers of impacts.
• LANCA^®^ model is assessing land use impacts on five soil properties and could be used in LCA.
• An updated version of the LANCA^®^ model is presented.
• Two aggregated multidimensional indices based on LANCA® model are proposed.
• Characterisation factors are provided for both land occupation and transformation.

## 1 Introduction

Soil quality degradation is the evident result of the increased pressure on land resources (MEA, 2005) associated with the intensification and expansion of human activities. Soil conservation is one of the main sustainability goals to ensure food security and environmental protection (Foley et al., 2011). Hence, it is crucial that methodologies assessing impacts caused by production and consumption of goods include land use related impacts in their frameworks. The United Nations Environmental Programme (UNEP) and the Society of Environmental Toxicology and Chemistry (SETAC) within the Life Cycle Initiative (UNEP-SETAC LC Initiative) have recommended incorporating the impact of the supply chain on soil quality in land use models (Curran et al., 2016). The incorporation of soil quality aspects is crucial in order for land use impact assessment to be more inclusive (Koellner et al., 2013a). However, this is challenged by the complexity of soil processes, as well as the spatial and temporal variability of soil properties (Li et al., 2007). This complexity leads to serious difficulties in defining a single soil quality indicator – or a minimum set of indicators – that represent a good compromise between guaranteeing a satisfying level of robustness in the assessment and avoiding overcomplicating the interpretation phase.

For these reasons, in the current Life Cycle Assessment (LCA) framework, soil properties and functions are still not systematically incorporated and evaluated. At the endpoint level, the damage caused to biodiversity has generally been the main target (e.g. species richness loss: De Baan et al., 2013a; Souza et al*.,* 2015). Alternatively, two main approaches can be found in the literature to address land use. The first is represented by land accounting, according to which the amount of land occupied and/or transformed for a certain activity is recorded. The second rely on midpoint models by quantifying the impacts in terms of variation of soil properties. The amount of land occupied and/or transformed are multiplied by characterisation factors reflecting soil properties changes for each type of land occupation and transformation. In these cases, the soil quality can be measured by means of a single indicator - e.g. soil organic matter (SOM) (Milà i Canals et al. 2007; Milà i Canals, 2007), soil organic carbon (Brandão & Milà i Canals, 2013), soil erosion (Núñez et al., 2013), salinisation (Payen et al., 2014) - or multiple indicators (e.g. the LANd use indicator value Calculation (LANCA^®^) model - Bos et al., 2016a; Saad et al., 2013; SALCA-SQ - Oberholzer et al., 2012). A model based on variation of SOM (Milà i Canals, 2007) due to land interventions is currently the one recommended by the Joint Research Centre of the European Commission (EC-JRC) for assessing land use impacts (EC-JRC, 2011; EC-JRC, 2012). Although SOM represents a crucial indicator of both the provisioning (e.g. biotic production) and the regulating ecosystems services (e.g. climate regulation), the risk related to adopting such a model is that other important soil functions are disregarded, e.g. soil resistance to erosion, filtration capacity etc., and some impacts are neglected e.g. compaction and salinisation (Mattila et al., 2011; Vidal Legaz et al., 2017). For a more detailed literature review on available land use impact assessment models at midpoint level, the reader is referred to Vidal Legaz et al. (2017).

Of the available models developed, LANCA^®^ (original model in Beck et al., 2010), as in the recent version released by Bos et al. (2016a), was selected as the recommended model for the impact evaluation in the Environmental Footprint (EF) framework (Sala et al., 2018). This model was originally developed to assess the impact of different interventions involving land use on five soil functions, i.e. biotic production (BP), groundwater recharge (GR), erosion resistance (ER), mechanical filtration (MF) and physiochemical filtration (PF), based on site specific data. The reasons for selecting this model for the EF framework were that:

- It follows a land use classification fully compatible with the International Life Cycle Data (ILCD) system
- It presents the highest coverage in terms of land use elementary flows (up to Level 4, according to the classification provided by Koellner et al., 2013b)
- It allows for global application of the characterisation and provides characterisation factors (CFs) both at global and country level
- It covers both occupation and transformation impacts
- It represents a robust attempt of modelling impacts on different soil properties and functions

Despite presenting several benefits, the orginal LANCA^®^ model has a number of drawbacks. Firstly, as it is composed by several coupled models, it is rather complex, and therefore the results it presents are difficult to communicate. Furthermore, as LCA is often used to compare the performance of different products, the drawback of a multi-indicator model is that, unless a method is suggested to aggregate the different indicators in a single score, the practitioner is left without an answer to determine the ranking of two product options (Vidal Legaz et al., 2017). Therefore, providing a single indicator for land use impact assessment would be ideal, especially considering that land use is just one of many impact categories covered by LCA.

This study aims at evaluating how the land use impact estimation provided by the LANCA^®^ model can be improved in terms of applicability from the practitioner perspective, without losing robustness from the methodological side. This objective was pursued namely in two ways: by selecting the most relevant indicators to avoid the use of redundant ones and by aggregating the selected indicators in a single index as comprehensive as possible. Even though, from a scientific point of view, there is no need for a single index as this may lead to a loss of information, a simplification would improve the easiness of the application and of the interpretation of the results. To this end, two alternative aggregation approaches are explored and the resulting indices are compared and discussed.

This paper is structured as follows. Section 2 illustrates the methodology adopted to create two aggregated indices based on the LANCA^®^ model. Section 3 reports the results of the two aggregation approaches, complemented with a discussion of the limitations of both the underlying model and the aggregated indices. The main findings and conclusions are presented in Section 4.

## 2 Methodology

The purpose of this section is to briefly introduce the LANCA^®^ model and to explain the methodology adopted towards the LANCA^®^ model refinement performed within in this study (Section 2.1).

Moreover, we present the three-step approach for the development of two aggregated indices to quantify the impacts on soil quality: the Soil Quality Index (SQI) and the Normalisation-based Soil Quality Index (NSQI).

The three steps are:

1. Comparison and statistical analyses of the CFs provided by the LANCA^®^ model for five soil impact indicators and selection of the most significant indicators to be used for calculating the aggregated indices (Section 2.2)
2. Re-scaling of the CFs following two alternative schemes (Section 2.3.1)
3. Aggregation of the selected indicators through two different schemes: one aggregating linearly the LANCA^®^ indicators and another based on normalisation integrated into the characterisation step (Section 2.3.2)

### 2.1 Description of the LANCA^®^ model and its refinement

The LANCA^®^ model provides a set of CFs for five different soil quality indicators both at global and at country level. When the country is not known the practitioner can use the global-default CFs, those are calculated as world averaged CFs based on the area of each country. The CFs can be used to characterise elementary flows related to land interventions (i.e. amounts of area and time of occupation for each land use type) provided in Life Cycle Inventories (LCI) (see Table 1), in terms of impacts on soil quality indicators. The starting point of this work was the version of the model (LANCA^®^ v2.3), presented in Bos et al. (2016b). Based on the work done in the EF context by EC-JRC, adopting and testing the LANCA^®^ v2.3 version, a refinement of the model has been proposed (as described in Section 2.1.1). This refinement underpins the LANCA^®^ v2.5 (Horn and Maier, 2018), as result of a cooperation between EC-JRC and the Fraunhofer Institute.

**Table 1:** LANCA^®^ impact indicators and related units (Bos et al., 2016a), fu: functional unit.

Indicator	Land use activity	LCI unit	CF unit	LCIA result unit
Erosion resistance	Occupation	m^2^*a/fu	kg soil/(m^2^*a)	kg soil loss
	Permanent transformation	m^2^/fu		(kg soil loss)/a
Mechanical filtration	Occupation	m^2^*a/fu	m^3^ water/(m^2^*a)	m^3^ reduced water infiltration
	Permanent transformation	m^2^/fu		(m^3^ reduced water infiltration)/a
Physicochemical filtration	Occupation	m^2^*a/fu	mol reduction potential/(m^2^)	(mol reduced physicochemical filtration capacity potential)*a
	Permanent transformation	m^2^/fu		(mol reduced physicochemical filtration capacity potential)
Groundwater regeneration	Occupation	m^2^*a/fu	m^3^ groundwater/(m^2^*a)	m^3^ reduced groundwater regeneration
	Permanent transformation	m^2^/fu		(m^3^ reduced groundwater regeneration)/a
Biotic production	Occupation	m^2^*a/fu	kg biotic production/(m^2^*a)	kg reduced biotic production
	Permanent transformation	m^2^/fu		(kg reduced biotic production)/a

The LANCA^®^ v2.5 version presents a number of elements of novelty compared to the previous one. The purpose of this section is to describe the latest update of the LANCA^®^ model (Section 2.1.1) and explain how the model deals with different types of land use activities (i.e. occupation and transformation) (Section 2.1.2). For more details on the general aspects of the LANCA^®^ model, the reader is referred to Bos et al. (2016a).

#### 2.1.1 Refinement of the LANCA^®^ model towards version 2.5

The CF for occupation of a specific land use type (*j*) in LANCA^®^ is calculated for each indicator (*i*) (e.g. biotic production) as the ecosystem quality (*Q*) difference between the reference situation and the respective chosen land use, as illustrated in Equation (1) (Bos et al., 2016a). Therefore, a land use activity associated with a low CF is expected to cause a small difference in the ecosystem quality compared to a situation in which it would not take place.

(1) $$CF_{occ,i,\;j}=Q_{i,\;ref}\;-Q_{i,\;j\;}$$

The term $Q_{i,\;j\;}$ is calculated by means of equations that take into account country-averaged parameters (e.g. average country slope), as explained in Bos et al. (2016a).

In LANCA^®^ v2.3, the reference situation of each country corresponded to the potential natural vegetation of the biome with the largest area share, based on the global distribution of biomes provided by Olson et al. (2001). This assumption caused a number of systematic inaccuracies in the model for large and/or heterogeneous countries. An example was the USA: due to its significant geographical spread, a wide range of biomes can be found in this country. However in the original model only one reference situation was considered, which was “Arctic or alpine tundra”, due to the fact that Alaska occupies almost 20% of the entire area of the country. As a consequence, in the calculation of the CFs for the occupation of arable land for the impact indicator biotic production, the resulting values provided in Bos et al. (2016b) for the USA were negative (i.e. suggesting that the use of arable land provides a benefit). This was because the level of biotic production of the reference situation adopted was lower than that of arable land, although most of the agricultural activities take place in warmer regions of the country, characterised by a different reference situation.

To increase the level of accuracy of the CFs, in LANCA^®^ v2.5 a different approach was adopted. This involved five procedural steps, as presented below.

- Step 1 - calculating the values of ecosystem quality for all the types of potential natural vegetation that can be found in a country according to the global map of ecological zones provided by FAO (2012).
- Step 2 – calculating the value of the reference situation ($Q_{i,\;ref}$) as a weighted average of the values above according to the area share of each ecological zone in a country considering all existing ecological zones
- Step 3 - repeating step 2 but excluding the following ecological zones in the calculation of the reference situation: “boreal tundra woodland”, “polar”, “subtropical desert”, “temperate desert” and “tropical desert”
- Step 4 – calculating CFs for all land use types excluding agricultural and forest-related land uses according to equation (1), using the value of reference situation calculated at step 2
- Step 5 – calculating CFs for agricultural and forest-related land uses according to equation (1), using the value of reference situation calculated at step 3

The reasoning behind this choice is that it is highly unlikely that agricultural or forestry activities will take place in a desert, tundra or polar area. In this way, many of the artificial outliers present in the previous version (v2.3) were removed. Nevertheless, according to FAO (2012), five countries are entirely belonging to either desert (i.e. Bahrain, Kuwait, Qatar and United Arab Emirates) or polar (i.e. Greenland) ecological zones. In such cases the correction described at steps 3 and 5 could not be applied and therefore the CFs provided for these countries need to be taken with care.

Another element of novelty to refine LANCA^®^ is the use of a new source for the global distribution of potential natural vegetation. In this version, the dataset previously used, Olson et al. (2001), has been replaced by a more updated one, FAO (2012). This alternative map of potential natural vegetation (PNV) was chosen as it was developed by harmonising existing datasets through a consultative method and is considered to be more reliable than Olson et al. (2001).

#### 2.1.2 Occupation and transformation impacts

An overview of the LANCA^®^ impact indicators and their related unit of measure for different land use activities is provided in Table 1. As can be seen from this table, the CFs provided have the same unit regardless the type of land use intervention (i.e. occupation, permanent transformation). As the inventory flow for land occupation records the area occupied (A) and the occupation time (T_occ_), while the inventory flow for land transformation only records the area occupied, the life cycle impact assessment (LCIA) results of land occupation (Eq. (2)) and transformation (Eq. (3)) are not directly additional in the case of permanent transformation (Koellner et al., 2013a).

(2) $$Occupation\;impact=\Delta Q\times T_{occ}\times A$$

(3) Permanenttransformationimpact=ΔQ×A

Where ΔQ is the difference in the ecosystem quality between the reference situation and the current (occupation impacts) or prospective (transformation impacts) land use. In both cases the CF is equal to ΔQ (as illustrated in Equation (1)).

In case of reversible transformation, according to Koellner et al. (2013a), the impact is calculated by taking into account the regeneration time (T_reg_) as illustrated by Equation (4). In this case, occupation and transformation impacts have the same unit of measure and, therefore, can be added together.

(4) Reversibletransformationimpact=ΔQ×Treg×0.5×A

The CF for reversible transformation is, therefore, calculated following Equation (5):

(5) $$CF\;\left(reversible\;transformation\right)=\Delta Q\times T_{reg}\times 0.5$$

Currently, the LANCA^®^ model only provides CFs for permanent transformations, hence, in order to account for reversible transformations, new CFs need to be calculated by assuming a regeneration time and following Equation (5).

The regeneration time depends on the intensity of the land use type during the transformation phase, on the impact pathway and on the ecosystem type (i.e. warm humid climates favour a faster regeneration in terms of biotic production but not in terms of erosion as they are more vulnerable to extreme climatic events) (Koellner et al., 2013a). Although there is limited knowledge on ecosystems regeneration times, a number of publications have proposed estimations of regeneration times (e.g. Muller-Wenk, 1998; Koellner and Scholz, 2007; van Dobben et al., 1998; Saad et al., 2013). The IPCC (2006) assumes that for biotic land uses the regeneration time is 20 years, although this is likely to be an underestimation of reality. Saad et al. (2013) suggest values of the regeneration time necessary for an ecosystem to recover to its maximum potential after clearance, according to each biome. These range from 52 years (for mangroves) to 138 years (for montane grassland and shrubland).

### 2.2 Statistical evaluation on characterisation factors

A statistical analysis of the correlation among the five indicators of the LANCA^®^ model was performed in order to select the most appropriate indicators in terms of relevance and unicity of information. Spearman's rank order correlation index (Sachs, 2012) was adopted to assess the statistical dependence of the global-default CFs provided for each indicator (according to the latest version of CFs, provided in Horn and Maier (2018)). The results of this analysis are reported in Table 2. The correlation analysis was performed with the occupation CFs, although the same applies to the “transformation to” CFs (as in the LANCA^®^ model they correspond to occupation CFs) and “transformation from” CFs (as they correspond to the occupation CFs taken with opposite sign). The purpose of this analysis was to identify how the ranking of land use types (in terms of their impact on the five indicators) was correlated across the indicators.

**Table 2:** Spearman's rank order correlation coefficients related to Occupation CFs (global averages). ER: erosion resistance; MF: mechanical filtration; PF: physicochemical filtration; GR: groundwater regeneration; BP: biotic production.

	ER	MF	PF	GR	BP
ER	1.00				
MF	−0.19	1.00			
PF	−0.19	1.00	1.00		
GR	−0.48	0.38	0.38	1.00	
BP	0.00	0.61	0.61	0.52	1.00

From this analysis it appeared that the indicator erosion resistance (ER) presented negligible correlation with all indicators excluding groundwater regeneration (in this case a low negative correlation was found). Groundwater regeneration (GR) resulted to have a low positive correlation with mechanical filtration (MF) and physicochemical filtration (PF) and a moderate positive correlation with biotic production (BP). This suggests that the information provided by ER and GR is quite specific and unlikely to be extrapolated from the other indicators. On the other hand, the indicators mechanical filtration (MF) and physiochemical filtration (PF) present a unitary correlation coefficient. This means that the information they provide may be considered redundant when ranking land use interventions, using global characterisation factors. The same is valid when land use interventions are ranked within the same country, in other words, for each country individually, the correlation between MF and PF of the different elementary flows is equal to one. Therefore, only one of these two indicators is retained in the aggregated index. Finally, the indicator biotic production (BP) presents a correlation coefficient of 0.61 with MF and PF indicators. Hence, the information carried by this indicator adds significant information not captured by the other indicators. The final selection of indicators was therefore:

- Erosion resistance
- Mechanical filtration
- Groundwater regeneration
- Biotic production

### 2.3 Aggregation of shortlisted indicators - two approaches towards a soil quality index

Once a number of indicators are selected, the complexity that is likely to arise from a multi-indicator model should be limited, thus simplifying the interpretation of the results. According to this, two aggregation approaches aimed at obtaining a single score index - enabling the quantification of impacts on soil quality - are presented: the Soil Quality Index (SQI) and the Normalisation-based Soil Quality Index (NSQI). Both indices provide a measure of the impact of different land use interventions on soil quality, hence higher values correspond to larger impacts.

The methodological differences in the development of the suggested indices took place either at the re-scaling phase or at the aggregation phase. Section 2.3.1 presents the re-scaling techniques adopted in each case, while Section 2.3.2 presents the aggregation approaches adopted in the development of the SQI and the NSQI.

#### 2.3.1 Re-scaling of the LANCA^®^ characterisation factors

In order to render indicators comparable prior to aggregation, they need to be normalised (JRC-OECD, 2008). Due to the specific meaning of the term “normalisation” within the LCA literature, in this article we will refer to the process of normalising indicators by using the term “re-scaling”.

As the re-scaling process can be affected by extreme values (i.e. outliers) that could become unintended benchmarks, the first step was to eliminate those values by:

- Identifying for each of the individual four indicators *i* (identified in Section 2.2) the value corresponding to the 5th (CF_i_^5^) and the 95th (CF_i_^95^) percentile of the distribution of country-specific CFs for “occupation” elementary flows *j.*
- Applying a cut-off to all the CFs smaller than CF_i_^5^ and larger than CF_i_^95^

The full list of cases (combinations of country and land use type) excluded by the cut-off criteria for each indicator is provided in the supporting information (SI). In this way, the practitioner can choose to use either the global set of CFs or the CFs of a country with similar climatic and geomorphological conditions if a case study in one of the countries affected by the cut-off has to be developed.

Then, two alternative re-scaling techniques were adopted.

The first (technique A) was used in the development of the SQI. According to this technique the re-scaled CFs were calculated following Equation (6). The resulting CFs are dimensionless and expressed as Points (Pt).

(6) $$\overset{˙}{CF_{i,j}}=\frac{CF_{i,j}}{CF_{i}^{95}}\;\times 100\;\left[Pt/m^{2}a\right]$$

Where:

$\overset{˙}{CF_{i,j}}$ is the re-scaled characterisation factor for the indicator *i* and the elementary flow *j*
$CF_{i,j}$ is the original characterisation factor for the indicator *i* and the elementary flow *j*
$CF_{i}^{95}\;$is the 95th percentile of the distribution of country-specific CFs for the indicator *i*

The second re-scaling technique (technique B) was used in the development of the NSQI. According to this technique the CFs were re-scaled following Equation (7).

(7) $$\overset{¨}{CF_{i,j}}=\frac{CF_{i,j}-CF_{i}^{5}\;}{CF_{i}^{95}-\;CF_{i}^{5}}\;\times 100\;\left[Pt/m^{2}a\right]$$

Where:

$\overset{¨}{CF_{i,j}}$ is the re-scaled characterisation factor for the indicator *i* and the elementary flow *j*
$CF_{i,j}$ is the original characterisation factor for the indicator *i* and the elementary flow *j*
$CF_{i}^{5}\;$ is the 5th percentile of the distribution of country-specific CFs for the indicator *i*
$CF_{i}^{95}\;$ is the 95th percentile of the distribution of country-specific CFs for the indicator *i*

**Fig. 1:**
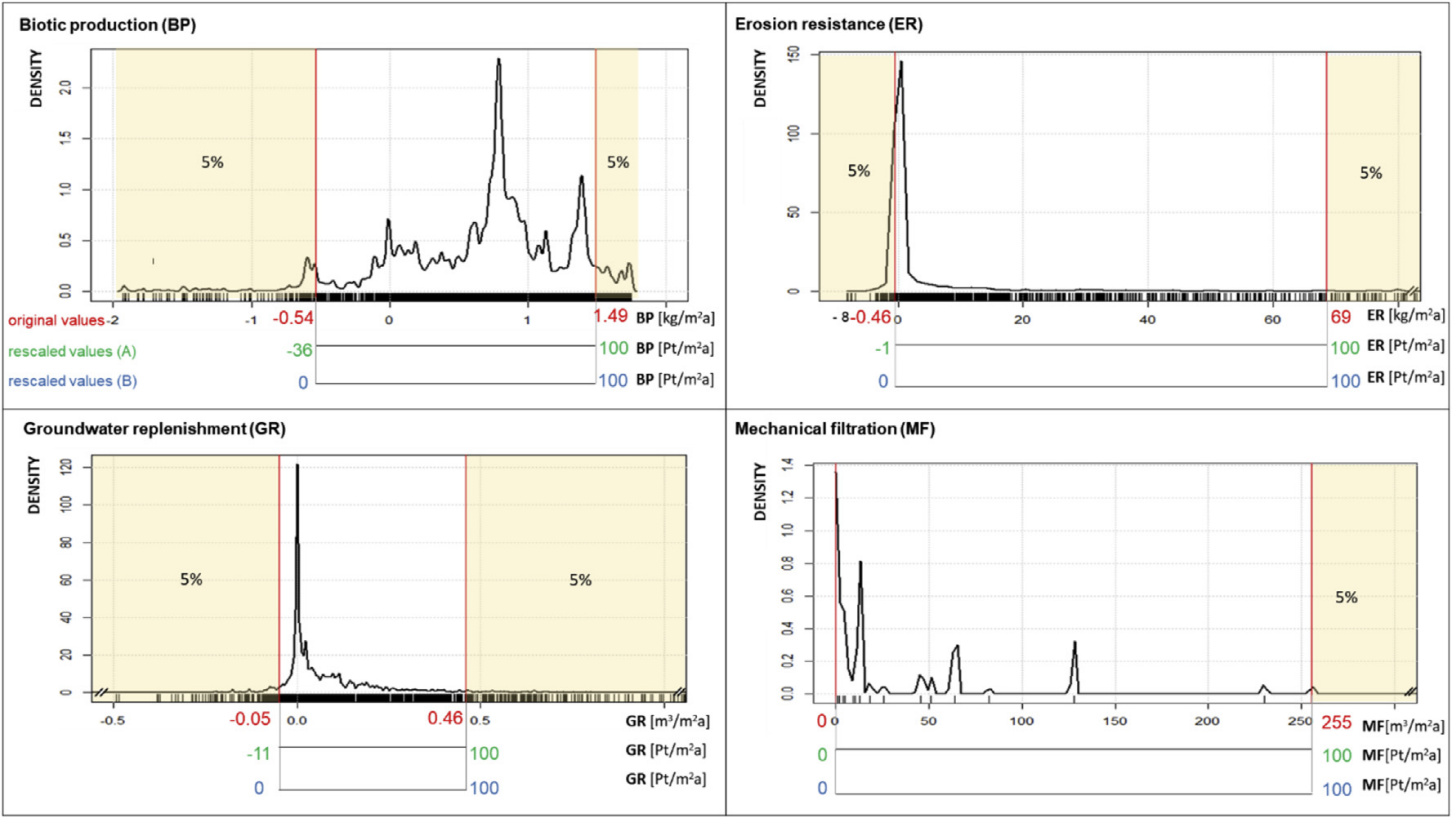
Visualisation of the re-scaling technique. Black line: kernel density estimation of the country-specific occupation CFs for all land use types. Values in red represent the 5th and 95th percentile of the distribution of the original CFs. Values in green are the re-scaled values according to technique A. Values in blue are the re-scaled values according to technique B. Yellow shaded areas: portion of CFs excluded by the applied cut-off. (For interpretation of the references to colour in this figure legend, the reader is referred to the Web version of this article.)

Fig. 1 provides a visualisation of the two re-scaling processes: the estimated probability density function of the country-specific CFs is represented for each indicator. The original values of the 5th and 95th percentile of the distribution of CFs are provided underneath the plot (in red). The re-scaled values according to technique A are provided underneath in green, and those calculated following technique B are provided below in blue. Table 3 reports the minimum and maximum of the distribution of original CFs, and of the two re-scaled sets of CFs, together with the values of the applied cut-offs.

**Table 3:** Overview of the two re-scaling techniques adopted. BP: biotic production; ER: erosion resistance; GR: groundwater regeneration; MF: mechanical filtration.

Indicator	Original values		Cutoff values		Re-scaled values			
					Technique A		Technique B	
	CF_MIN_	CF_MAX_	CF^5th^	CF^95th^	CF_MIN_	CF_MAX_	CF_MIN_	CF_MAX_
BP	−1.93	1.75	−0.54	1.49	−36	100	0	100
EP	−8.15	624.9	−0.46	68.57	−1	100	0	100
GR	−1.17	1.74	−0.05	0.46	−11	100	0	100
MF	0	1149.75	0	255.5	0	100	0	100

The conceptual difference between the two re-scaling techniques lies in the way they deal with CFs presenting negative signs for land occupation activities (i.e. reporting a benefit deriving from land occupation). The first re-scaling technique was designed in order to maintain the original sign that the CFs have in the LANCA^®^ model and to ensure that a CFs equal to zero would remain such when re-scaled. The second re-scaling technique was designed in order to ensure that all “occupation” CFs would be positive (or zero), to avoid that the calculation of normalisation references could lead to negative values.

#### 2.3.2 Description of the aggregation approaches adopted

At this step, two single point indices were developed, each representing a new set of CFs.

The Soil Quality Index was obtained by aggregating the “Occupation” CFs re-scaled according to technique A (described in Section 2.3.1). In this case, the CFs relative to each indicator *i* were added together using equal weights (1-1-1-1) in order to obtain one single number for each elementary flow (Equation (8)). The new set of CFs for occupation thus obtained ranged from −17 to 165 for the global CFs and from −47 and 318 for the country-specific CFs. The soil quality index CFs are expressed in Points per unit of inventory flow (Pt/m^2^a).

(8) $$\bar{\;CF_{occ,j}}=\sum_{i=1}^{4}\dot{CF_{ij}}\;\left[Pt/m^{2}a\right]$$

Where:

$\bar{\;CF_{occ,j}}$ is the aggregated SQI CF for the occupation of land use type *j* expressed in Pt/m^2^a
$\overset{˙}{CF_{ij}}$ is the re-scaled occupation CF for the indicator *i* and elementary flow *j* calculated in Equation (6) expressed in Pt/m^2^a

The Normalisation-based Soil Quality Index (NSQI), was developed by applying normalisation factors to each shortlisted indicator and aggregating the normalised scores using equal weights. Country-specific normalisation references, namely total national land use impacts, were calculated and used to normalise the CFs from the LANCA^®^ model re-scaled according to technique B (Section 2.3.1). According to this, the resulting unit of the characterisation is a weighted result expressed in points per unit of inventory flow (Pt/m^2^a). The normalisation references used in this work correspond to the occupation impacts calculated using the re-scaled CFs (according to technique B) and the globally-applicable national land use inventories developed in Faragò et al. (2018). A dimensionless correction factor was additionally applied to remove the effect of the country size on the normalisation reference, which would then be reflected as part of the new set of CFs. To this purpose, the normalisation references or national impacts from land use were divided by a factor (α) equal to the total land area of each country divided by the total world land area. A full list of normalisation references is reported in the SI.

The reason for using the re-scaled CFs rather than the original CFs provided by LANCA^®^, is that the normalisation references calculated with the original set of CFs would have provided for some countries negative values, making the normalisation process hard to interpret.

Taking the selected four indicators *i* (see Section 2.2), the calculation described in Equation (9) was applied to derive aggregated NSQI CFs, thus reducing the four sets of occupation CFs to one single set. For the purpose of this paper, equal weighting between land use impact indicators has been adopted, as assumed in the first approach (SQI).

(9) $$\bar{\;CF_{occ,j}}=\overset{4}{\underset{i=1}{\sum }}\frac{\overset{¨}{CF_{ij\;}}}{NR_{i}}\;\times \;\propto \;\left[Pt/m^{2}a\right]$$

Where:

$\bar{\;CF_{occ,j}}$ is the aggregated NSQI CF for the occupation of land use type *j*, expressed in Pt/m^2^a.
$\overset{¨}{CF_{ij\;}}$ is the re-scaled occupation CF for the indicator *i* and elementary flow *j* calculated in Equation (7), expressed in Pt/m^2^a
$NR_{i}$ is the normalisation reference for the impact indicator *i* expressed in Pt
α is the country size correction factor

The new set of CFs for occupation thus obtained ranged between 1.06E-14 and 1.47E-13 Pt/m^2^a for the global CFs and between 6.66E-16 and 3.89E-11 Pt/m^2^a for the country-specific CFs.

An advantage of the NSQI approach over the SQI is the removal of modelling uncertainties, as these would affect in the same way the characterisation scores in the numerator and the normalisation reference in the denominator, thus leaving only the uncertainties related to the input parameters of the model. A numerical example illustrating how both aggregated indices are calculated is presented in the SI.

#### 2.3.3 Calculation of CFs for reversible transformations

Characterisation factors for transformation impacts were calculated as in Saad et al. (2013), following Equation (5), by assuming a regeneration time of 20 years for biotic land uses and of 85 years for artificial land use types (sealed land), following Brandão and Mila i Canals (2013). Therefore according to both approaches (SQI and NSQI), CFs for transformation for all land use types were calculated following equations (10), (11)).

(10) $$\bar{CF_{transf,\;\;to}}=\bar{\;CF_{occ}}\;\times 0.5\;\times \;T_{reg}\;\left[Pt/m^{2}\right]$$

(11) $$\bar{CF_{transf,\;\;from}}=\;-\;\bar{\;CF_{occ}}\times 0.5\;\times \;T_{reg}\;\left[Pt/m^{2}\right]$$

$\bar{CF_{transf,\;\;to}}\;$ is the aggregated CF for “transformation to” expressed in Pt/m^2^
$\bar{CF_{transf,\;\;from}}\;$ is the aggregated CF for “transformation from” expressed in Pt/m^2^

## 3 Results and discussion

The following subsections present the results of each aggregation approach (Sections 3.1, 3.2). A comparison between the two approaches follows in Section 3.3 and the limitations and critical assumptions of both the LANCA^®^ model and the aggregated models are discussed in Section 3.4. The two sets of CFs calculated both at global and country level, for land occupation and transformation flows are provided in the SI.

### 3.1 Approach 1: Soil Quality Index

**Fig. 2:**
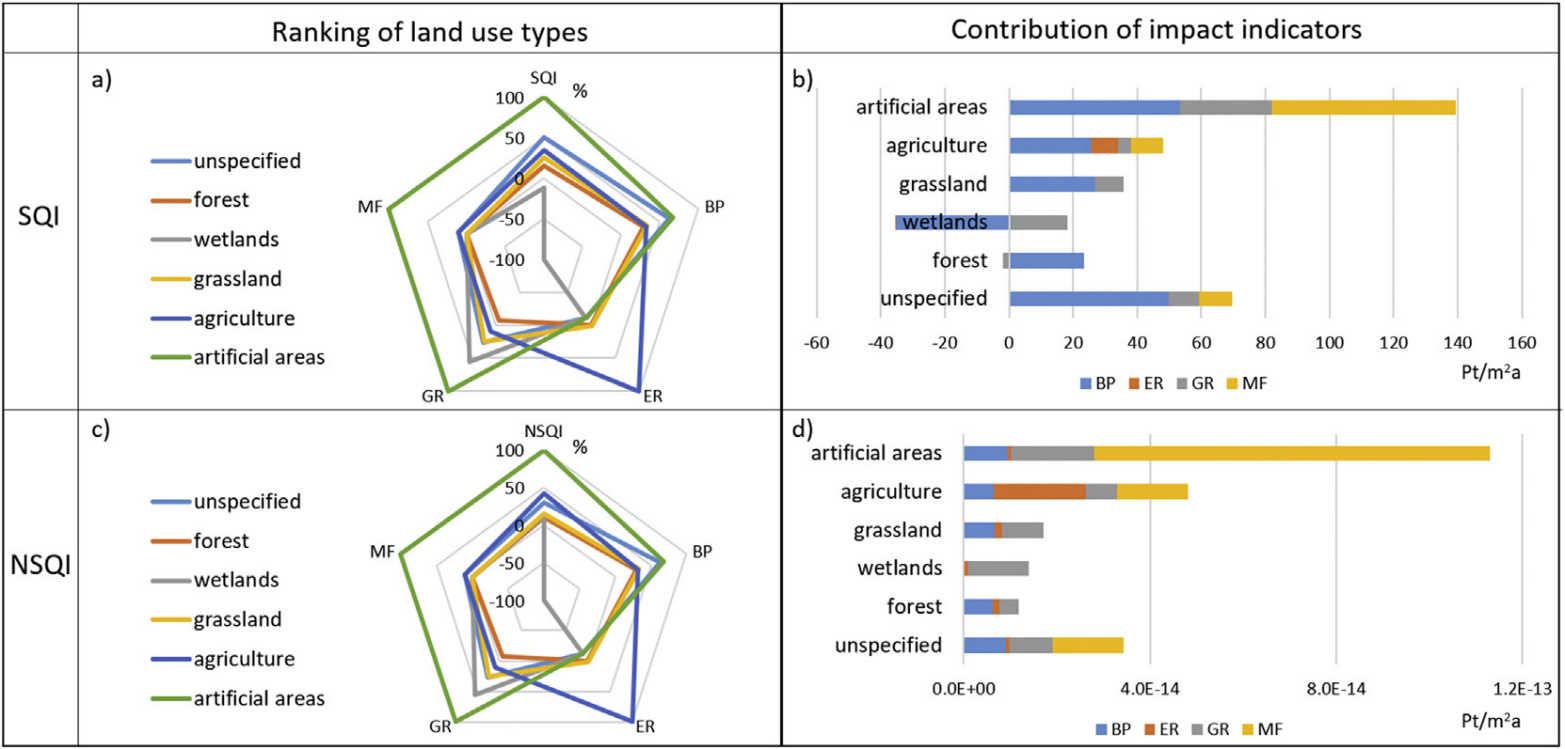
Comparison between the global SQI (a) and NSQI (c) CFs and the global CFs calculated for the four selected LANCA^®^ indicators for land occupation (BP, ER, GR,MF). In each case, the highest CF (absolute value) is taken as a reference (i.e. 100% or −100%) and the others are expressed as percentage with reference to it. Contribution of each indicator to the global SQI (b), and NSQI (d) obtained for six land use types (values are in Pt/m^2^a). BP: biotic production; ER: erosion resistance; GR: groundwater regeneration; MF: mechanical filtration; SQI: soil quality index; NSQI: normalisation-based soil quality index.

Fig. 2-a shows a comparison between the global CFs provided by Horn and Maier (2018) for a selection of six land use types and the SQI obtained for each land use type (for a detailed description of the classification of land use types please refer to Koellner et al., 2013b). It is possible to see that artificial areas are assigned the highest value of SQI (equal to 139 Pt/m^2^a), having the highest CFs across all impact indicators other than erosion resistance. This is due to the fact that artificial areas have a high sealing factor (i.e. a parameters used in the calculation of the mechanical filtration and the biotic production indicators that describes the degree of surface sealing caused by different land uses). In contrast, wetlands present the lowest CFs for all impact indicators other than groundwater regeneration, and consequently present the lowest SQI (−17 Pt/m^2^a). In this case, the negative value indicates a potential improvement against the reference situation. The contribution of each impact indicator to the soil quality index obtained for the selected land use types is presented in Fig. 2-b. The contribution of the different indicators to the total index varies according to the land use type: for artificial areas, mechanical filtration is the predominant indicator, while for the remaining land use types the predominant indicator is biotic production. Finally, the indicator erosion resistance is contributing to the aggregated index mostly for agricultural land occupation.

**Fig. 3:**
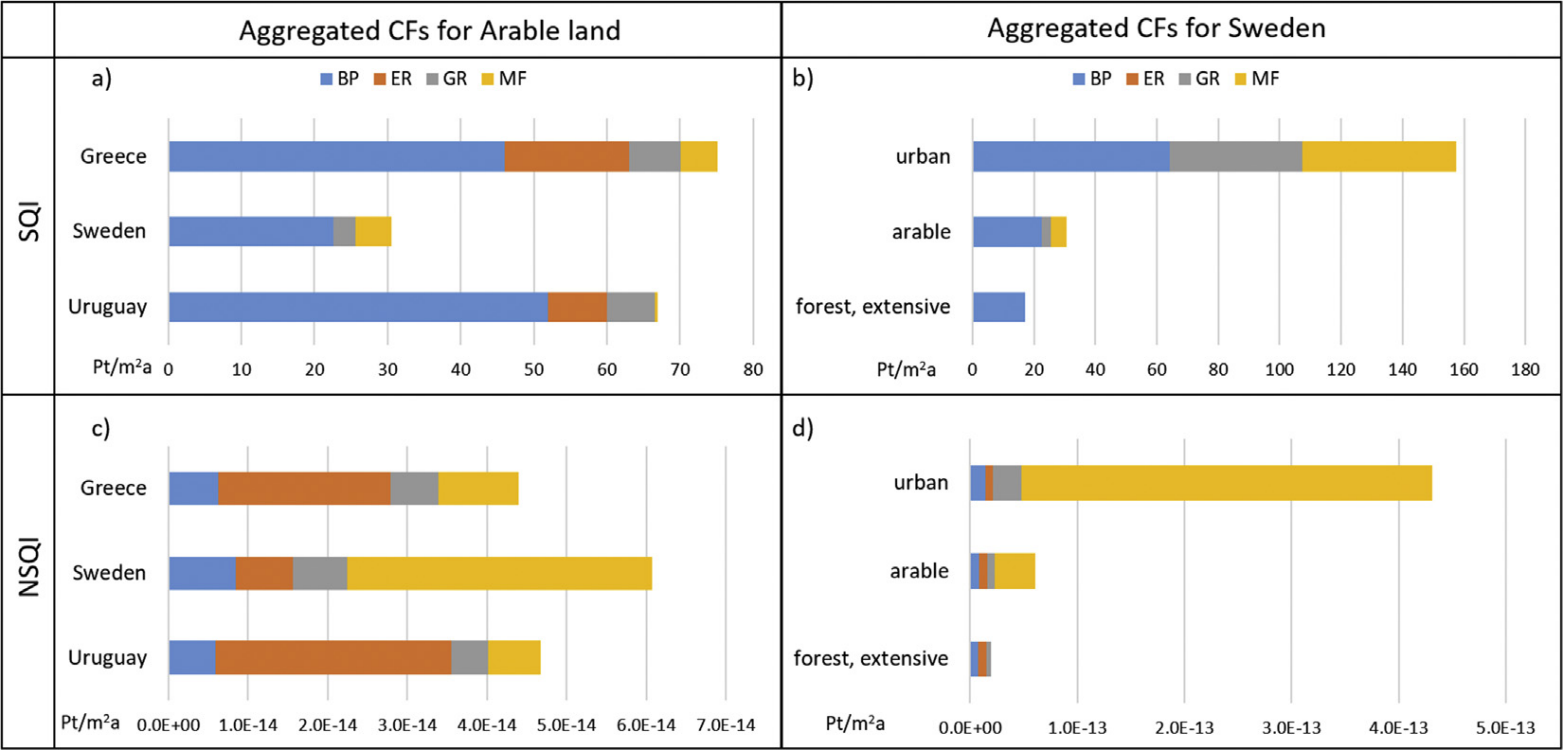
Contribution of each indicator to the SQI (a) and NSQI (c) CFs for arable land occupation in Greece, Sweden, and Uruguay. Contribution of each indicator to the SQI (b) and NSQI (d) CFs for occupation of urban land, arable land, and extensive forest in Sweden. All values are in Pt/m^2^a. BP: biotic production; ER: erosion resistance; R: groundwater regeneration; MF: mechanical filtration; SQI: soil quality index; NSQI: normalisation-based soil quality index.

At country level, a similar analysis can be performed both across land use types and across countries. This is presented in Fig. 3-a and 3-b. Fig. 3-a shows the contribution of each impact indicator to the soil quality index obtained for the three selected countries for arable land occupation; it illustrates that although Uruguay and Greece present similar values of soil quality index for arable land occupation (respectively 67 Pt/m^2^a and 75 Pt/m^2^a), in the first case this is mainly caused by a relatively higher value of biotic production (contributing to almost 80% of the total index), while in the second the contribution of the category erosion resistance is also significant (contributing to more than 20% of the total index). This demonstrates that the soil quality index is able to reflect country specific differences in the relative share of a driver of soil quality impact compared to another. Fig. 3-b provides a comparison across land use types, similar to the one presented at global level, considering one country (i.e. Sweden): it is possible to see that the ranking of land use types for this country is consistent with the one found at global level. Furthermore, it presents the contribution of each impact indicator to the soil quality index obtained for the three land use types, showing how for this country the impact indicator biotic production is predominant for all three land use types considered.

### 3.2 Approach 2: Normalisation-based Soil Quality Index

A comparison between the global CFs provided by the LANCA^®^ model for a selection of six land use types and the normalisation-based soil quality index obtained for each land use type, both referring to occupation flows is presented in Fig. 2-c. Similarly, to the case of the SQI, artificial areas are assigned the highest value of NSQI. However, in this case, the lowest value of the aggregated index was assigned to forest occupation. The contribution of each impact indicator to the NSQI obtained for the selected land use types is presented in Fig. 2-d. It is worth stressing that the leading role of the indicator biotic production encountered for the SQI was not found in this case, while the indicator mechanical filtration ended up being by large the main contributor to the aggregated index for artificial areas (responsible for 75% of the total value).

A similar analysis was conducted at country level, and is presented in Fig. 3-c and 3-d. Fig. 3-c shows a comparison between the NSQI obtained for occupation of arable land in three selected countries, and the contribution of each impact indicator to the NSQI. It is possible to see that with this aggregation approach, the indicator erosion resistance becomes predominant for Uruguay and Greece, while the indicator mechanical filtration has the largest contribution for Sweden. Fig. 3-d provides a comparison across land use types, similar to the one presented at global level, considering one country (i.e. Sweden). A similar trend to the one found with the SQI is visible, with urban land use presenting the highest value of NSQI, followed by arable land and ultimately by extensive forest. When considering the contribution of each impact indicator to the NSQI the indicator mechanical filtration is predominant for both urban and arable land occupation, while in case of forest occupation the predominant indicator is biotic production.

### 3.3 Comparison between the two approaches

A comparison between the global CFs obtained with the two indices for a selection of six land use types is presented in Fig. 2. It is possible to observe that in all cases, artificial areas are associated with the highest CF. There are some differences in the ranking of land use types according to the two indices, although the overall trend is similar. When assessing the contribution of the different LANCA^®^ indicators to the aggregated indices, it is possible to notice that the indicator mechanical filtration is more predominant in the NSQI CFs than in the SQI CFs for artificial areas, agriculture and unspecified land occupation. This is expected, as these land use types have a relatively lower contribution to the normalisation references of the other impact categories – i.e. groundwater regeneration and biotic production – than the one found in the case of mechanical filtration. Hence, BP, ER and GR will result in being less significant than MF in the contribution analysis of the resulting aggregated CFs. Due to similar reasons, the indicator erosion resistance presents a higher contribution in the NSQI CFs than in the SQI CFs for agricultural land.

**Fig. 4:**
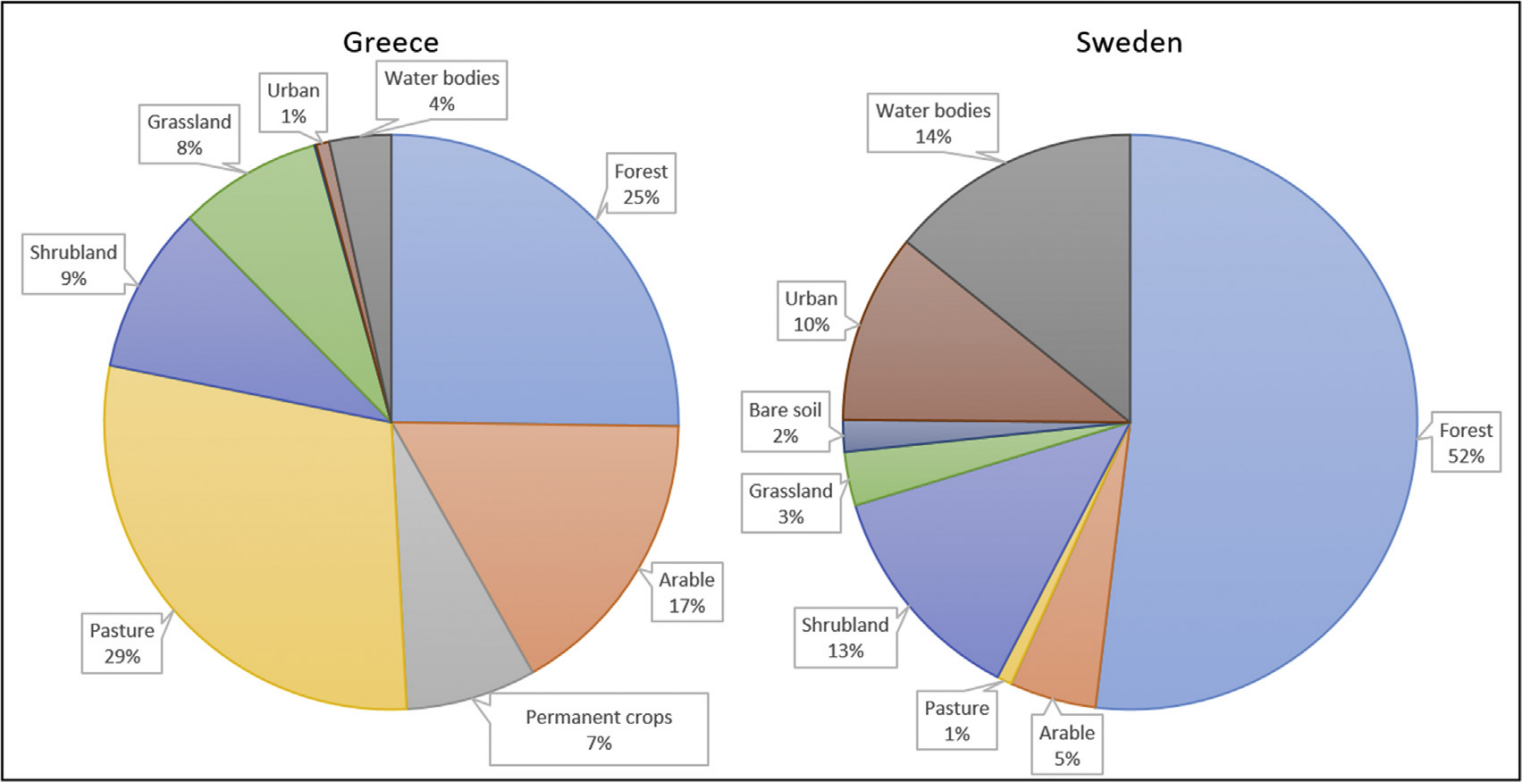
Land cover in Greece and Sweden according to Faragó et al. (2019).

Fig. 3 presents a comparison between the two indices across three countries (Greece, Sweden and Uruguay) for one land use type – arable land - (Fig. 3-a and 3-c) and across three land use types (urban, arable and extensive forest) for one country – Sweden - (Fig. 3-b and 3-d). It is interesting to note how the ranking of countries varies between the SQI (where Sweden presents the lowest value) and the NSQI (where Sweden presents the highest); this is mainly due to the variation of the relative contribution of the indicator mechanical filtration. This is reasonable as country-specific conditions determine the CFs calculated with the NSQI aggregation approach. Therefore, in the case of Sweden, where more than half of the surface is currently covered by forests (Faragó et al., 2019), the area-adjusted normalisation reference for mechanical filtration is lower (making the normalised indicator higher) compared to Greece, which is currently covered by a lower share of forests (25%) and a higher share of arable land (17%) (Fig. 4). When instead one country is selected (in this case Sweden), the ranking of land use types is similar across the two approaches; however, due to differences in the re-scaling and aggregation approaches adopted, the relative contribution of the four indicators to the aggregated index changes from one approach to another.

In parallel, an analysis was conducted to evaluate how the 212 countries assessed by LANCA^®^ were ranked according to each index for selected land use types. From this analysis it appeared that the SQI and the NSQI show very different ranking of countries (the correlation between the ranking of countries in the SQI and in the NSQI was 0.35 for arable land and −0.06 for artificial areas). This is in line with what was found for the three countries represented in Fig. 3 and is reasonable when considering that with the NSQI approach the aggregated CFs are determined by country-specific conditions (as illustrated with the example of Greece and Sweden).

To investigate which impact indicators were driving the values of the aggregated indices in each approach, a Spearman-rank-order correlation test was performed between the two aggregated sets of global CFs (SQI and NSQI) and each impact indicator. The results are presented in Table 4. It is possible to see how all indicators apart from erosion resistance present an either moderate or high positive correlation with the SQI. The indicator biotic production presents the highest positive correlation (0.86), while the indicator erosion resistance presents negligible correlation with the index (0.06). The low correlation between erosion resistance and the SQI is caused by the fact that a number of land use types present high CFs for all impact indicators, and therefore also for the SQI, except for erosion resistance (e.g. artificial areas, urban, traffic area). This is supported also by the results presented in Table 2, where it is possible to see that the indicator erosion resistance had a negative or negligible correlation with all the other LANCA^®^ indicators. The underlying reasons of the apparent different behaviour of this indicator compared to the others and its implications are discussed more in depth in Section 3.4.1. The high correlation (0.81) between mechanical filtration and the SQI supports the decision to omit the indicator physicochemical filtration when calculating the aggregated index. Had this indicator been retained, the aggregated index would have been driven mainly by these two indicators combined (the correlation between both mechanical filtration and physicochemical filtration and a variation of the SQI calculated considering all five indicators is 0.85).

**Table 4:** Spearman-rank correlation between the single impact indicators and the two aggregated indices (SQI, and NSQI) using global average CFs. BP: biotic production; ER: erosion resistance; GR: groundwater regeneration; MF: mechanical filtration; SQI: Soil Quality Index; NSQI: Normalisation-based Soil Quality Index.

	ER	MF	GR	BP
SQI	0.06	0.81	0.54	0.86
NSQI	0.13	0.83	0.46	0.73

The correlation between the NSQI and the impact indicators is presented in Table 4; similarly to what seen for the SQI all indicators are positively correlated with the aggregated index, with erosion resistance presenting a negligible correlation (0.13) and mechanical filtration the highest correlation (0.83).

To conclude, Table 4 clearly shows that in both the SQI and the NSQI the results of the aggregation are mainly driven by the biotic production and mechanical filtration indicators. This is reasonable when considering that these are the two indicators with the highest correlation (equal to 0.61 as illustrated in Table 2). A risk of over-representation of these two soil properties as opposed to, e.g. erosion resistance, cannot be ruled out, and for this reason in a future development of this work an alternative weighting approach could be explored designed to adjust for this imbalance.

### 3.4 Limitations and critical assumptions

The following subsections provide an in depth discussion of the limitations of both the LANCA^®^ model and the aggregation approaches suggested.

#### 3.4.1 Limitations of the LANCA^®^ characterisation model

As this work builds on the LANCA^®^ model, which is still being refined, a number of limitations of the model also affect the aggregated indices.

Firstly, the level of accuracy in the calculation of the CFs presents room for improvement. Currently, CFs are calculated as explained in Section 2.1.1 by applying Equation (1) for each combination of country and land use type. To take into account the spatial distribution of the soil property parameters, this calculation would have to be performed using a fully integrated geographic information system (GIS) - based approach. Thus, LANCA^®^ would be calculated for a global raster of 1 km cell size. An improved procedure would entail:

- Calculating the global quality map of each land use type ($Q_{i,\;j})$ and indicator
- Calculating the global quality map of the reference situation based on the potential natural vegetation at cell level ($Q_{i,\;ref})$ for each indicator
- Deriving occupation CF maps based on Equation (1) $(CF_{occ,i,\;j}$)
- Calculating a weighted average of the CFs at country level, by taking into account only the cells where that specific land use activity is currently taking place (according to current datasets of land cover)

Such an approach would ensure that CFs are calculated only considering the specific conditions of the areas where an activity is most likely to take place. For instance, in the case of Egypt, according to current land cover maps (Jun et al., 2014), agricultural activities take place almost uniquely along the river Nile. It is therefore logic that the term $Q_{i,\;j\;}$ (for agricultural land uses), should be calculated using parameters specific for that area, instead of averaged at country level. Furthermore, a similar logic should be applied when calculating global CFs, that are currently calculated as a weighted average of the country-specific CFs, based on the total area of each country. Instead, it would be preferable to take into account the current land cover, by considering for each country the area where a certain land use activity takes place, rather than the area of the entire country in defining the weights.

Secondly, in the version of LANCA^®^ used in this work (v2.5) the CFs provided for the class “forest, natural”, which according to the definition by Koellner et al. (2013a) is “forest not used by humans”, should by logic be equal to zero for all those countries in which the potential natural vegetation is forest in the entire country. However, this is not always true: in the case of Singapore (PNV 100% tropical forest) the value of biotic production of “forest, natural” is 0.95 kg/m^2^a, while in the case of Faroe Islands (PNV 100% boreal coniferous forest) the same value is equal to zero. From this, it is clear that the land class “forest, natural” is modelled in LANCA^®^ as a coniferous forest in all cases, while it should be modelled considering the site-specific natural forest in each location.

Furthermore, in the calculation of the indicator biotic production, country-specific conditions are not taken into account, and the CFs vary only with the land use type and the chosen reference situation. As this soil function can be linked to the indicator soil organic carbon (SOC) as illustrated by Boone et al. (2018), and there is availability of global SOC databases at a high resolution (e.g. ESDAC (2012) database), the modelling of this soil function could be improved using available data sources. An alternative could be to replace the indicator biotic production directly with SOC.

The distributions of CFs presented in Fig. 1 suggest that for all indicators a part from biotic production, the LANCA^®^ model generates a number of extreme values (identified as outliers) that are most likely caused by a number of modelling limitations. It is therefore suggested that in a future development of LANCA^®^, specific cases characterised by such extreme values should be investigated in detail to further refine the model. A starting point of this could be the list of cases affected by the applied cut-off criteria provided in the SI.

When it comes to modelling erosion resistance, the LANCA^®^ model is affected by a limitation deserving attention and possible future refinement of the model. As the model only considers erosion caused by rainfall, assessed using the Revised Universal Soil Loss Equation (Renard et al., 1997), it fails to take into account the anthropogenic net soil loss that takes place when natural land is converted to urban land. Additionally, it considers that sealed land (e.g. roads and urban land in general) is less vulnerable to soil erosion, attributing to the occupation of these types of land a negative CF for erosion (i.e. a benefit). This is conceptually debatable, as sealing land, although making it less vulnerable to soil erosion from rainfall, represents a very invasive type of land use in terms of soil conservation. For this reason we believe the LANCA^®^ model should be revised to provide CFs for occupation of/transformation to artificial land uses for erosion resistance that are calculated taking this anthropogenic soil loss into account.

This last limitation of LANCA^®^ is the reason behind the negative correlation of ER with the other four indicators (Table 1) and the negligible correlation of ER with the SQI and NSQI (Table 4). If these correlations are re-calculated without taking artificial land uses into account the correlation coefficient between ER and the SQI is equal to 0.67, while the correlation coefficient between ER and the NSQI is equal to 0.88. This entails that the ER indicator is well represented in the indices proposed for all the remaining land use types. This is expected to become true also for urban land uses once the issue identified with the modelling in LANCA^®^ of soil erosion in urban areas is solved.

#### 3.4.2 Limitations of the re-scaling techniques

The methods followed to create the aggregated indices involved the re-scaling of the original CFs provided by LANCA^®^ (to ensure that the resulting CFs would be comparable across all impact indicators as illustrated in Fig. 1), according to two re-scaling techniques. To avoid that the re-scaling process would be affected by the presence of extreme values and/or outliers, in both cases, the extreme values were removed by the application of a cut-off. Although this entails loosing part of the information carried by the original model, we believe the values removed by the cut-offs to be artificial outliers (i.e. extreme values caused by modelling limitations). In a future development of the aggregated indices, we consider investigating alternative methods to re-scale the CFs, such as for example using categorical scales in which a score is assigned to each CF based on its percentile within the distribution of CFs (JRC-OCED, 2008). The benefit of this method is that it is not affected by outliers, the drawback is that part of the information would be lost (i.e. CFs belonging to the same group would be assigned the same score even though they might have different values in the original set). Finally, this method would not enable to maintain the original sign of the CFs in the re-scaling process (as is currently the case in the first re-scaling technique suggested).

#### 3.4.3 Limitations of aggregation approaches

A critical assumption of both proposed aggregation approaches is the choice of applying equal weighting when calculating the aggregated indices. Several weighting techniques are available, some derive from statistical models and take into consideration the redundancy of the indicators (e.g. principal component analysis, factor analysis), others rely on participatory methods based on experts’ judgement (e.g. budget allocation process) (JRC-OECD, 2008). For instance, in both aggregation approaches, different weights could be attributed to the four impact indicators after consulting experts on their level of significance and irreversibility. For example, since impacts of erosion are less reversible than those of a reduction in groundwater recharge, a greater weight could be assigned to the former indicator. However, this would also require taking into account the spatial differences of both significance and irreversibility of each indicator. Alternatively, literature sources may perhaps help gauge the importance of the different categories of land use impacts between each other. Although at this stage, for the sake of simplicity, it was decided not to take this path, a more refined choice of the weights to be assigned in the aggregation process is envisaged in a future development of this work.

The choice of removing the indicator PF from the aggregated indices, motivated by the linear relationship found between the indicators MF and PF in terms of ranking of land use interventions within a country or when using the global-default CFs (illustrated in Section 2.2), presents a drawback. These two indicators result uncorrelated when they are compared in terms of how they rank different countries for the same land use type. In other words the variation of the PF across countries cannot be extrapolated from the variation of MF across countries and, therefore, part of the information carried by the PF might be lost due to this methodological choice. For a more detailed explanation of this analysis the reader is referred to the SI.

For what concerns the NSQI approach, uncertainties related to the national inventories need to be considered, as these are quite country-specific as addressed in Faragó et al. (2018). Furthermore, inventories may gain more accuracy in the future if the differentiation on land classes increases. At the moment only a part of the available land use types from the LANCA^®^ model has been used used for the computation of the normalisation references, mainly leaving out the refined, disaggregated land use types (e.g. arable land irrigated, arable land non irrigated). Finally, a drawback of the NSQI approach is that the calculation of the normalisation references depends on a global inventory of land use activities, which is influenced by the year to which the inventory refers. Therefore, the resulting CFs should be regularly updated to accommodate potential large changes in land use at country level.

## 4 Conclusion and outlook

Current models for land use impact assessment in LCA suffer from either a lack of comprehensiveness in modelling the key drivers of impacts on soil quality or an excessive complexity and site-specificity, which limits their applicability in LCA. To fill this gap in research, this work critically assessed the strengths and weaknesses of the LANCA^®^ model, which had been selected as the recommended model to assess land use impacts within the Environmental Footprint framework. Moreover, we proposed two approaches for calculating a Soil Quality Index, in order to improve the model applicability and interpretation.

Throughout this process, a detailed assessment of the original set of characterisation factors was conducted which enabled the identification of a number of shortfalls in the LANCA® model version 2.3, leading to a model refinement in version 2.5. Additionally, a number of limitations of the model that still need to be tackled are presented, including:

1. The use of averaged country parameters (instead of adopting a higher resolution) in the calculation of country-specific characterisation factors
2. Modelling limitations that cause a number of extreme values amongst the characterisation factors (i.e. artificial outliers), and
3. The lack of characterisation factors to be used for reversible land transformation that already include considerations on the regeneration time.

Future development of the LANCA^®^ model should adopt a higher resolution in the calculation of country-specific characterisation factors, and use land cover datasets to derive representative country-averaged and world-averaged characterisation factors, as suggested in Section 3.4.1. In this way, we envision that most of the outliers identified in this work will be eliminated.

The last shortfall identified was partially overcome in this work by calculating characterisation factors for reversible land transformation for the two aggregated indices; nevertheless, the regeneration times applied are likely to be an underestimation of reality and further research is needed to derive regeneration time estimates for different ecosystem types and impact pathways (Saad et al., 2013).

Two main novel elements for midpoint land use life cycle impact assessment are presented in this paper. Firstly, two sets of CFs are provided for a large range of land use types (i.e. up to Level 4, according to the classification by Koellner et al., 2013b) that enable assessing the impacts of land use on four soil properties addressed by the LANCA^®^ model (biotic production, erosion resistance, groundwater regeneration and mechanical filtration) synthetized in one aggregated index. The CFs are provided as global default and country-specific values for both occupation and transformation land use interventions. Secondly, two approaches are presented for calculating the aggregated index: the soil quality index (SQI), which is based on linear aggregation and is a potential candidate for the assessment of land use impacts within the Environmental Footprint and the normalisation based soil quality index (NSQI), based on aggregating the indicators after normalising them against absolute normalisation references. Both approaches use equal weighting across the indicators. The two aggregation approaches suggested in this work represent a potential way forward towards the widespread application of a comprehensive and robust land use model at midpoint level in LCA.

Of the two alternative indices presented in this article, we believe priority should be given to LCA case study applications of the SQI, while the NSQI could be used to conduct a sensitivity analysis to evaluate the influence of the different aggregation approaches over the results. Furthermore, given that there are several orders of magnitudes between the CFs calculated with the NSQI approach and the SQI approach, comparative case study applications can shed light on what the ideal magnitude and variability of the CFs are to ensure that in the characterisation step the relevance of the inventory is not overestimated.
