# Supplementary material for: Soil quality index: Exploring options for a comprehensive assessment of land use impacts in LCA
Source: J Clean Prod. 2019 Apr 1;215:63–74. doi: 10.1016/j.jclepro.2018.12.238 (PMC6472660; doi:10.1016/j.jclepro.2018.12.238)
Supplement: Multimedia component 1 [file mmc1.docx]

**Supplementary Materials**

**Soil quality index: exploring options for a comprehensive assessment of land use impacts in LCA**

Valeria De Laurentiis^1^, Michela Secchi^1^, Ulrike Bos^2^, Rafael Horn^2^, Alexis Laurent^3^, Serenella Sala^1^*

^1^European Commission, Joint Research Centre,Via Enrico Fermi 2749, I-21027 Ispra (VA), Italy

^2^*University of Stuttgart, Insitute for Acoustics and Building Physics , Department Life Cycle Engineering, Wankelstrasse 5, D-70563 Stuttgart*

^3^ *Technical University of Denmark (DTU),Division for Quantitative Sustainability Assessment (QSA), Department of Management Engineering, Lyngby, Denmark*

* Corresponding author: [serenella](mailto:michela.secchi@ext.ec.europa.eu).sala@ec.europa.eu

Telephone: +39 0332 786417

Contents

[Tables of outliers 1](#_Toc529877054)

[Relationship between mechanical filtration and physicochemical filtration 9](#_Toc529877055)

[Example of calculation of the aggregated indices 10](#_Toc529877056)

[Soil quality index characterisation factors 12](#_Toc529877057)

[Normalisation-based soil quality index characterisation factors 12](#_Toc529877058)

[References 13](#_Toc529877059)

# Tables of outliers

This section reports, for each indicator separately, all the countries for which one or more characterisation factors (CFs) were affected by the cut-off applied in the re-scaling process, as presented in Section 2.3.1 of the article. For each country, the total number of elementary flows excluded is listed, together with the level 1 land use class to which they belong according to the classification by Koellner et al. (2013) (0 = unspecified, 1 = forest, 2 = wetlands, 3 = shrubland, 4 = grassland, 5 = agriculture, 6 = agriculture-mosaic, 7 = artificial areas, 8 = bare area, 9 = snow and ice).

Additionally the overall share of outliers is provided. This is equal to the total number of outliers divided by the total number of CFs for each indicator (equal to 12084 = 57 flows X 212 countries). Finally, the area-weighted overall share is provided (in which the area of each country is taken into account when calculating the share of outliers).

The purpose of reporting these tables of outliers is to inform the practitioner that in the countries reported below and for the flows indicated, by applying the soil quality index, he/she might be underestimating/overestimating the contribution of one (or more) impact indicators.

**Table 1** Biotic production: number of outliers for each country and level 1 land use class to which they belong.

| **Country** | **Number of outliers** | **Level 1 land use class** |
| --- | --- | --- |
| Bahrain | 29 | 1,2,5,6 |
| Bangladesh | 7 | 7 |
| Belize | 12 | 7 |
| Brazil | 12 | 7 |
| Brunei Darussalam | 16 | 0,7,8,9 |
| Cameroon | 12 | 7 |
| Congo | 16 | 0,7,8,9 |
| Congo DRC | 16 | 0,7,8,9 |
| Costa Rica | 4 | 7 |
| Cote d'Ivoire | 16 | 0,7,8,9 |
| Dominica | 16 | 0,7,8,9 |
| Equatorial Guinea | 16 | 0,7,8,9 |
| Fiji | 16 | 0,7,8,9 |
| French Guiana | 16 | 0,7,8,9 |
| Gabon | 16 | 0,7,8,9 |
| Ghana | 12 | 7 |
| Greenland | 29 | 1,2,5,6 |
| Grenada | 16 | 0,7,8,9 |
| Guadeloupe | 16 | 0,7,8,9 |
| Guyana | 16 | 0,7,8,9 |
| Haiti | 12 | 7 |
| Honduras | 3 | 7 |
| Indonesia | 16 | 0,7,8,9 |
| Jamaica | 16 | 0,7,8,9 |
| Kuwait | 29 | 1,2,5,6 |
| Liberia | 16 | 0,7,8,9 |
| Malaysia | 16 | 0,7,8,9 |
| Martinique | 16 | 0,7,8,9 |
| Montserrat | 3 | 7 |
| New Caledonia | 16 | 0,7,8,9 |
| Nicaragua | 16 | 0,7,8,9 |
| Panama | 16 | 0,7,8,9 |
| Papua New Guinea | 12 | 7 |
| Philippines | 16 | 0,7,8,9 |
| Puerto Rico | 16 | 0,7,8,9 |
| Qatar | 29 | 1,2,5,6 |
| Saint Kitts and Nevis | 4 | 7 |
| Saint Lucia | 16 | 0,7,8,9 |
| Saint Vincent and the Grenadines | 16 | 0,7,8,9 |
| Sao Tome and Principe | 16 | 0,7,8,9 |
| Sierra Leone | 16 | 0,7,8,9 |
| Singapore | 16 | 0,7,8,9 |
| Solomon Islands | 16 | 0,7,8,9 |
| Sri Lanka | 7 | 7 |
| Suriname | 16 | 0,7,8,9 |
| Trinidad and Tobago | 16 | 0,7,8,9 |
| Turks and Caicos Islands | 16 | 0,7,8,9 |
| United Arab Emirates | 29 | 1,2,5,6 |
| Vanuatu | 16 | 0,7,8,9 |
| Overall share | **10%** | |
| Area weighted overall share | **7%** | |

**Table 2** Erosion potential: number of outliers for each country and level 1 land use class to which they belong.

| **Country** | **Number of outliers** | **Level 1 land use class** |
| --- | --- | --- |
| Afghanistan | 19 | 0,2,3,7 |
| Andorra | 2 | 8,9 |
| Angola | 19 | 0,2,3,7 |
| Australia | 19 | 0,2,3,7 |
| Azerbaijan | 19 | 0,2,3,7 |
| Bhutan | 46 | 0,1,2,5,6,7,8,9 |
| British Virgin Islands | 2 | 8,9 |
| Brunei Darussalam | 4 | 7,8,9 |
| Cameroon | 2 | 8,9 |
| Chad | 19 | 0,2,3,7 |
| Chile | 19 | 0,2,3,7 |
| China | 19 | 0,2,3,7 |
| Colombia | 18 | 2,5,7,8,9 |
| Comoros | 28 | 2,5,6,7,8,9 |
| Costa Rica | 35 | 0,2,5,6,7,8,9 |
| Djibouti | 21 | 0,2,3,7 |
| Dominica | 15 | 5,7,8,9 |
| Dominican Republic | 2 | 8,9 |
| Ecuador | 36 | 0,2,5,6,7,8,9 |
| El Salvador | 19 | 5,7,8,9 |
| Equatorial Guinea | 7 | 5,7,8,9 |
| Eritrea | 19 | 0,2,3,7 |
| Fiji | 19 | 5,7,8,9 |
| French Guiana | 2 | 8,9 |
| Gabon | 2 | 8,9 |
| Grenada | 4 | 7,8,9 |
| Guatemala | 22 | 2,5,7,8,9 |
| Guinea | 4 | 7,8,9 |
| Guyana | 2 | 8,9 |
| Haiti | 7 | 5,7,8,9 |
| Honduras | 19 | 5,7,8,9 |
| India | 19 | 0,2,3,7 |
| Indonesia | 22 | 2,5,7,8,9 |
| Iran | 19 | 0,2,3,7 |
| Iraq | 19 | 0,2,3,7 |
| Israel | 19 | 0,2,3,7 |
| Jamaica | 2 | 8,9 |
| Kyrgyzstan | 23 | 0,2,3,7,8,9 |
| Laos | 28 | 2,5,6,7,8,9 |
| Liberia | 2 | 8,9 |
| Liechtenstein | 24 | 2,5,6,7,8,9 |
| Madagascar | 2 | 8,9 |
| Malaysia | 24 | 2,5,6,7,8,9 |
| Martinique | 4 | 7,8,9 |
| Mayotte | 4 | 7,8,9 |
| Mexico | 19 | 0,2,3,7 |
| Montserrat | 4 | 7,8,9 |
| Morocco | 19 | 0,2,3,7 |
| Myanmar | 22 | 2,5,7,8,9 |
| Namibia | 19 | 0,2,3,7 |
| Nepal | 40 | 0,1,2,5,6,7,8,9 |
| New Caledonia | 5 | 5,7,8,9 |
| New Zealand | 24 | 2,5,6,7,8,9 |
| Nicaragua | 2 | 8,9 |
| Pakistan | 19 | 0,2,3,7 |
| Panama | 19 | 5,7,8,9 |
| Papua New Guinea | 36 | 0,2,5,6,7,8,9 |
| Peru | 36 | 0 2 3 5 7 8 9 |
| Philippines | 28 | 2,5,6,7,8,9 |
| Puerto Rico | 4 | 7,8,9 |
| Rwanda | 2 | 8,9 |
| Saba | 5 | 5,7,8,9 |
| Saint Eustatius | 2 | 8,9 |
| Saint Lucia | 4 | 7,8,9 |
| Saint Vincent and the Grenadines | 19 | 5,7,8,9 |
| Sao Tome and Principe | 35 | 0,2,5,6,7,8,9 |
| Sierra Leone | 4 | 7,8,9 |
| Solomon Islands | 35 | 0,2,5,6,7,8,9 |
| Sri Lanka | 4 | 7,8,9 |
| Thailand | 2 | 8,9 |
| Timor-Leste | 22 | 2,5,7,8,9 |
| Tunisia | 19 | 0,2,3,7 |
| United States | 19 | 0,2,3,7 |
| Vanuatu | 28 | 2,5,6,7,8,9 |
| Venezuela | 4 | 7,8,9 |
| Vietnam | 7 | 5,7,8,9 |
| Yemen | 19 | 0,2,3,7 |
| **Overall share** | **10%** | |
| **Area weighted overall share** | **11%** | |

**Table 3** Groundwater regeneration: number of outliers for each country and level 1 land use class to which they belong.

| **Country** | **Number of outliers** | **Level 1 land use class** |
| --- | --- | --- |
| Albania | 1 | 7 |
| Andorra | 8 | 7 |
| Anguilla | 49 | 0,2,4,5,6,7,8,9 |
| Aruba | 49 | 0,2,4,5,6,7,8,9 |
| Austria | 7 | 7 |
| Bahamas | 16 | 0,2,7,8,9 |
| Bangladesh | 13 | 2,7,8,9 |
| Belize | 8 | 7 |
| Bhutan | 37 | 1,3,5,6,7 |
| Bonaire | 49 | 0,2,4,5,6,7,8,9 |
| Brazil | 8 | 7 |
| British Virgin Islands | 49 | 0,2,4,5,6,7,8,9 |
| Brunei Darussalam | 19 | 2,4,7,8,9 |
| Cambodia | 8 | 7 |
| Cameroon | 1 | 7 |
| Cayman Islands | 16 | 0,2,7,8,9 |
| Chile | 8 | 1,3 |
| Colombia | 21 | 1,2,3,7,8,9 |
| Comoros | 1 | 7 |
| Costa Rica | 27 | 1,2,3,4,7,8,9 |
| Dominica | 13 | 2,7,8,9 |
| Ecuador | 16 | 1,3,7 |
| El Salvador | 8 | 7 |
| Equatorial Guinea | 8 | 7 |
| Faroe Islands | 13 | 2,7,8,9 |
| Fiji | 13 | 2,7,8,9 |
| French Guiana | 13 | 2,7,8,9 |
| Gabon | 8 | 7 |
| Gambia | 16 | 0,2,7,8,9 |
| Gibraltar | 28 | 0,2,4,5,7,8,9 |
| Greenland | 44 | 0 1 3 4 5 6 7 |
| Grenada | 7 | 7 |
| Guatemala | 8 | 7 |
| Guinea | 8 | 7 |
| Guinea-Bissau | 8 | 7 |
| Guyana | 8 | 7 |
| Iceland | 16 | 1,3,7 |
| Indonesia | 19 | 2,4,7,8,9 |
| Ireland | 1 | 7 |
| Jamaica | 1 | 7 |
| Japan | 8 | 7 |
| Kenya | 8 | 7 |
| Laos | 21 | 1,2,3,7,8,9 |
| Liberia | 13 | 2,7,8,9 |
| Liechtenstein | 8 | 7 |
| Malaysia | 19 | 2,4,7,8,9 |
| Martinique | 8 | 7 |
| Montenegro | 8 | 7 |
| Myanmar | 21 | 1,2,3,7,8,9 |
| Nepal | 9 | 1,3,7 |
| New Caledonia | 7 | 7 |
| New Zealand | 8 | 7 |
| Nicaragua | 8 | 7 |
| Norway | 8 | 1,3 |
| Pakistan | 1 | 7 |
| Panama | 13 | 2,7,8,9 |
| Papua New Guinea | 27 | 1,2,3,4,7,8,9 |
| Peru | 9 | 1,3,7 |
| Philippines | 19 | 2,4,7,8,9 |
| Saba | 8 | 7 |
| Saint Barthelemy | 28 | 0,2,4,5,7,8,9 |
| Saint Eustatius | 8 | 7 |
| Saint Kitts and Nevis | 7 | 7 |
| Saint Martin | 28 | 0,2,4,5,7,8,9 |
| Saint Pierre and Miquelon | 8 | 7 |
| Saint Vincent and the Grenadines | 8 | 7 |
| Sao Tome and Principe | 13 | 2,7,8,9 |
| Sierra Leone | 13 | 2,7,8,9 |
| Singapore | 13 | 2,7,8,9 |
| Sint Maarten | 28 | 0,2,4,5,7,8,9 |
| Slovenia | 8 | 7 |
| Solomon Islands | 28 | 0,2,4,5,7,8,9 |
| South Korea | 8 | 7 |
| Sri Lanka | 8 | 7 |
| Suriname | 8 | 7 |
| Switzerland | 8 | 7 |
| Thailand | 8 | 7 |
| Timor-Leste | 8 | 7 |
| Trinidad and Tobago | 8 | 7 |
| Turks and Caicos Islands | 8 | 7 |
| US Virgin Islands | 28 | 0,2,4,5,7,8,9 |
| Vanuatu | 19 | 2,4,7,8,9 |
| Vatican City | 28 | 0,2,4,5,7,8,9 |
| Venezuela | 8 | 7 |
| Vietnam | 8 | 7 |
| **Overall share** | **10%** | |
| **Area weighted overall share** | **3%** | |

**Table 4** Mechanical filtration: number of outliers for each country and level 1 land use class to which they belong.

| **Country** | **Number of outliers** | **Level 1 land use class** |
| --- | --- | --- |
| Afghanistan | 10 | 7 |
| Algeria | 10 | 7 |
| Andorra | 10 | 7 |
| Argentina | 10 | 7 |
| Austria | 10 | 7 |
| Belgium | 10 | 7 |
| Bosnia and Herzegovina | 10 | 7 |
| Canarias | 10 | 7 |
| Chad | 10 | 7 |
| Chile | 10 | 7 |
| China | 10 | 7 |
| Comoros | 10 | 7 |
| Croatia | 10 | 7 |
| Czech Republic | 10 | 7 |
| Egypt | 10 | 7 |
| Faroe Islands | 10 | 7 |
| France | 10 | 7 |
| Iceland | 10 | 7 |
| Ireland | 10 | 7 |
| Jersey | 10 | 7 |
| Libya | 10 | 7 |
| Liechtenstein | 10 | 7 |
| Mauritania | 10 | 7 |
| Mexico | 10 | 7 |
| Montenegro | 10 | 7 |
| Morocco | 10 | 7 |
| Namibia | 10 | 7 |
| Nepal | 10 | 7 |
| New Zealand | 10 | 7 |
| Niger | 10 | 7 |
| North Korea | 10 | 7 |
| Norway | 10 | 7 |
| Pakistan | 10 | 7 |
| Peru | 10 | 7 |
| Portugal | 10 | 7 |
| Saudi Arabia | 10 | 7 |
| Serbia | 10 | 7 |
| Slovakia | 10 | 7 |
| Slovenia | 10 | 7 |
| South Africa | 10 | 7 |
| Sudan | 10 | 7 |
| Switzerland | 10 | 7 |
| The Former Yugoslav Republic of Macedonia | 10 | 7 |
| Tunisia | 10 | 7 |
| United States | 10 | 7 |
| Vatican City | 10 | 7 |
| Yemen | 10 | 7 |
| Zimbabwe | 10 | 7 |
| **Overall share** | **4%** | |
| **Area weighted overall share** | **5%** | |

# Relationship between mechanical filtration and physicochemical filtration

The following figures provide a visual representation of the relationship between mechanical filtration (MF) and physicochemical filtration (PF) indicators in LANCA® discussed in Section 2.2 of the article.

Figure 1 and Figure 2 illustrate respectively how these two indicators vary across land use types for the set of global average CFs and for the set of CFs provided for Italy. It is possible to see that in both cases the indicators MF and PF have a linear relationship (presenting a correlation coefficient equal to one). This means that according to these two indicators land use types are assigned the same ranking (from the highest to the lowest impact) within a country, and when the global-average CFs are used.

Figure 3 shows how the indicators MF and PF vary across the 212 countries for one chosen land use type (i.e. occupation of arable land). It is possible to see that in this case the two indicators are uncorrelated. In other words the variation of the indicator PF across countries cannot be explained by looking at the variation of the indicator MF. Hence, when excluding the indicator PF from the aggregated indices, this part of the information carried by the indicator PF is lost.

Figure 1: Relationship between mechanical filtration (MF) and physicochemical filtration (PF) indicators across land use types for global average values

Figure 2: Relationship between mechanical filtration (MF) and physicochemical filtration (PF) indicators across land use types for Italy

Figure 3: Relationship between mechanical filtration (MF) and physicochemical filtration (PF) indicators across countries for arable land

# Example of calculation of the aggregated indices

A numerical example is provided below to illustrate step by step how the SQI and NSQI CFs were calculated for both occupation and transformation impacts. The procedure will be illustrated using as an example the calculation of the CF for arable land in Uruguay (the term *j* in the equations below) with the two aggregation approaches.

Firstly, the LANCA CFs for this elementary flow *j* are:

CF_BP,j_ = 0.773 kg/m^2^a

CF_ER,j_ = 5.541 kg/m^2^a

CF_GR,j_ = 0.030 m^3^/m^2^a

CF_MF,j_ = 1.004 m^3^/m^2^a

All these values fall within the interval delimited by the 5^th^ percentile and the 95^th^ percentile of the respective distributions of CFs, and are therefore not affected by the applied cut-off.

Procedure for the calculation of the SQI

Equation 6 in the main text is applied to calculate the re-scaled CFs according to technique A. The values at the denominator of Equation 6 are provided for each indicator in Table 3 of the manuscript. This results in:

$\dot{{CF}_{BP,j}}=\frac{0.77}{1.49} \times100=52$ Pt/m^2^a

$\dot{{CF}_{ER,j}}=\frac{5.54}{68.57} \times100=8.1$ Pt/m^2^a

$\dot{{CF}_{GR,j}}=\frac{0.03}{0.46} \times100=6.6$ Pt/m^2^a

$\dot{{CF}_{MF,j}}=\frac{1.00}{255.5} \times100=0.39$ Pt/m^2^a

The resulting index (reported in Figure 3a) is calculated according to Equation 8

$\bar{{CF}_{occ,j}}={52+8.1+6.6+0.39=67}$ Pt/m^2^a

The CFs for reversible transformation are then calculated assuming a regeneration time of 20 years by means of Equation 10 and 11.

$\bar{{CF}_{transf, to}}=67 \times0.5 \times20=670$ Pt/m^2^

$\bar{{CF}_{transf, from}}=- 67 \times0.5 \times20= -670$ Pt/m^2^

Procedure for the calculation of the NSQI

Equation 7 in the main text is applied to calculate the re-scaled CFs according to technique B. The values of the 5th and 95th percentile of the distributions of CFs are provided for each indicator in Table 3 of the manuscript. This results in:

$\ddot{{CF}_{BP,j}}=\frac{0.77-(-0.54)}{1.49- (-0.54)} \times100=65$ Pt/m^2^a

$\ddot{{CF}_{ER,j}}=\frac{5.54-(-0.46)}{68.57- (-0.46)} \times100=8.6$ Pt/m^2^a

$\ddot{{CF}_{GR,j}}=\frac{0.03-(-0.05)}{0.46- (-0.05)} \times100=16$ Pt/m^2^a

$\ddot{{CF}_{MF,j}}=\frac{1.00-0}{255.5- 0} \times100=0.39$ Pt/m^2^a

The normalisation references for each indicator (${NR}_{i}$ in Equation 9) and the country size correction factor (α in Equation 9) for Uruguay, are provided in the spreadsheet attached below for the NSQI. These are:

${NR}_{BP}=$ 1.11E+13 Pt

${NR}_{ER}=$ 2.95E+11 Pt

${NR}_{GR}=$3.41E+12 Pt

${NR}_{MF}=$6.13E+10 Pt

α = 1.02E-03

The resulting index (reported in Figure 3b) is then calculated according to Equation 9.

$\bar{{CF}_{occ,j}}={(\frac{65}{1.11E+13}}+\frac{8.6}{2.95E+11}+\frac{16}{3.41E+12}+\frac{0.39}{6.13E+10}) \times$ 1.02E-03 = 4.67E-14 Pt/m^2^a

The CFs for reversible transformation are then calculated assuming a regeneration time of 20 years by means of Equation 10 and 11.

$\bar{{CF}_{transf, to}}=\text{4.67E-14}\times0.5 \times20=\text{4.67E-13}$ Pt/m^2^

$\bar{{CF}_{transf, from}}=\text{- }\text{4.67E-14} \times0.5 \times20= \text{- }\text{4.67E-13}$ Pt/m^2^

# Soil quality index characterisation factors

This spreadsheet provides:

- The original LANCA CFs across all 5 indicators for occupation flows
- The aggregated soil quality index CFs for occupation, transformation to and transformation from flows
- Histograms showing the contribution of each indicator to the total SQI, for the global set of occupation CFs and for two selected countries

# Normalisation-based soil quality index characterisation factors

This spreadsheet provides:

- The original LANCA CFs across all 5 indicators for occupation flows
- The re-scaled LANCA CFs across all 5 indicators for occupation flows
- The normalisation references calculated for all 5 indicators for occupation flows and the country size correction factors
- The aggregated normalisation-based soil quality index CFs for occupation, transformation to and transformation from flows
- Histograms showing the contribution of each indicator to the total NSQI, for the global set of occupation CFs and for two selected countries

# References

Koellner T., de Baan L., Beck T., Brandao M., Civit B., Goedkoop M., Margni M., Milà i Canals L., Müller-Wenk R., Weidema B., Wittstock B. (2013b). Principles for life cycle inventories of land use on a global scale. Int J Life Cycle Assess (2013) 18:1203–1215.
